# TRENTOOL: A Matlab open source toolbox to analyse information flow in time series data with transfer entropy

**DOI:** 10.1186/1471-2202-12-119

**Published:** 2011-11-18

**Authors:** Michael Lindner, Raul Vicente, Viola Priesemann, Michael Wibral

**Affiliations:** 1 Center for Economics and Neuroscience, University Bonn, Bonn, Germany; 2 Center for Individual Development and Adaptive Education of Children at Risk (IDeA), Frankfurt, Germany; 3 Dept. Neurophysiology, Max Planck Institute for Brain Research, Frankfurt, Germany; 4 Frankfurt Institute for Advanced Studies (FIAS), Frankfurt, Germany; 5 Dept. Neural Systems and Coding, Max Planck Institute for Brain Research, Frankfurt, Germany; 6 Group of Neural Theory, Ecole Normale Superieure, Paris, France; 7 MEG Unit, Brain Imaging Center, Goethe University, Frankfurt, Germany

## Abstract

**Background:**

Transfer entropy (TE) is a measure for the detection of directed interactions. Transfer entropy is an information theoretic implementation of Wiener's principle of observational causality. It offers an approach to the detection of neuronal interactions that is free of an explicit model of the interactions. Hence, it offers the power to analyze linear and nonlinear interactions alike. This allows for example the comprehensive analysis of directed interactions in neural networks at various levels of description. Here we present the open-source MATLAB toolbox TRENTOOL that allows the user to handle the considerable complexity of this measure and to validate the obtained results using non-parametrical statistical testing. We demonstrate the use of the toolbox and the performance of the algorithm on simulated data with nonlinear (quadratic) coupling and on local field potentials (LFP) recorded from the retina and the optic tectum of the turtle (Pseudemys scripta elegans) where a neuronal one-way connection is likely present.

**Results:**

In simulated data TE detected information flow in the simulated direction reliably with false positives not exceeding the rates expected under the null hypothesis. In the LFP data we found directed interactions from the retina to the tectum, despite the complicated signal transformations between these stages. No false positive interactions in the reverse directions were detected.

**Conclusions:**

TRENTOOL is an implementation of transfer entropy and mutual information analysis that aims to support the user in the application of this information theoretic measure. TRENTOOL is implemented as a MATLAB toolbox and available under an open source license (GPL v3). For the use with neural data TRENTOOL seamlessly integrates with the popular FieldTrip toolbox.

## Background

Making predictions is the essence of science. We sum up our experimental observations in hypotheses about causal interactions. To this end, causality has been conceptualized in the experimental sciences by making use of manipulations and predictions: If we manipulate the state of a part of the system in various ways (e.g. using stimuli or direct intervention) and can predict the outcome of each manipulation for another other part of the system (e.g. the neurophysiological responses) in the form of probabilities we say that the manipulation was causal to the outcome (see [1,2] for a more formal account). Despite the successful use of this concept in neuroscience, the self-generated activity of the brain poses a fundamental challenge. Due to this activity, we frequently observe a rather large variability of responses despite constant stimuli [3]. In addition, it is difficult to infer causality for the case of completely internally generated dynamics where there is no controlled experimental manipulation, e.g. when investigating the dynamics of the resting state. A deliberate manipulation of self generated activity is extremely difficult by definition. Hence, we have to loosen our requirements for ascribing causality to be able to also investigate directed interactions in systems with self generated dynamics. One popular way of augmenting the concept of causality was introduced by Norbert Wiener [4]. In Wiener's definition an improvement of the prediction of the future of a time series *X *from its own past by the incorporation of information from the past of a second time series *Y *is seen as an indication of a causal interaction from *Y *to *X*. Despite Wiener's use of the word causality in this context, this concept is today more often referred to either as predictive information flow [5] or Wiener-Akaike-Granger-Schweder influence [6], reflecting the progress made in the rigorous formulation of causal dependencies [1,2]. Here, we will use the term 'directed interaction' when referring to a property of the system under investigation - the 'ground truth', and we will use 'predictive information flow' in the context of metrics that indicate such directed interactions.

So far most implementations of Wiener's principle used model based approaches^1^. The earliest practical realization by Granger for example modeled the interacting parts of a system as autoregressive and their coupling as linear [7]. However, in a complex system - such as the brain - nonlinear behavior of its parts and nonlinear interactions between them have to be expected. In fact nonlinear phase-to-amplitude and amplitude-to-amplitude interactions between frequencies are reported frequently [8-10]. Non-linear interactions can take an unlimited number of different forms (e.g. quadratic, sigmoidal or step functions,..) - in contrast to linear ones. Hence, the type of interaction will usually be unknown and we cannot construct a suitable model of the interaction. To exhaustively cover all the possible types of non-linear interactions in the brain, and thereby to fully map the neural networks of interest, it would be useful to implement Wiener's principle in a way that is free of a model of the interaction (also see [11]).

Indeed, it is possible to reformulate Wiener's principle based on information theoretic quantities to reach the desired model-freeness. The resulting measure was originally formulated by Schreiber [12] and termed *transfer entropy *(TE). Shortly after its publication TE found first applications to neurophysiological data [13]. However, it was not until the introduction of new, data efficient estimators [14,15] that TE has experienced a rapid surge of interest [10,11,16-26]. Applications of TE in neuroscience comprise recordings in cultured neuronal populations [18], invasive electrophysiological recordings [26], magneto- and electroen-cephalography (MEG/EEG) [11,27], functional magnetic resonance imaging (fMRI) [21] and interactions between electrophysiological and fMRI signals [23]. Despite widespread interest in the method, no publicly available toolbox for neural data exists^2 ^that guides the user through the difficulties of this powerful, yet admittedly complex, technique.

TRENTOOL (the TRansfer ENtropy TOOLbox (Additional File 1)) fills this gap for the neurosciences by bundling data efficient estimation algorithms with the necessary parameter estimation routines and nonparametric statistical testing procedures for comparison between experimental conditions or groups of subjects.

The remainder of this manuscript is organized as follows. We first describe the toolbox and its use. Next, we give a detailed description of the definition and computation of TE as it is implemented in the toolbox. Two further sections demonstrate the performance of the toolbox for simulated data and a neurophysiological test case. We close by discussing merits and potential pitfalls of TE analysis and highlight the differences between TRENTOOL and other toolboxes for TE estimation.

## Implementation

This section describes the TRENTOOL toolbox first from the user's perspective - with a subsection explaining the use of TRENTOOL with different analysis strategies in mind. These different analysis strategies motivate several auxiliary routines that TRENTOOL provides to make TE estimation and statistical testing easier. These routines are then explained in depth in the second subsection, together with a definition of TE and a detailed description of its computation.

### Using TRENTOOL

**Figure 1:**
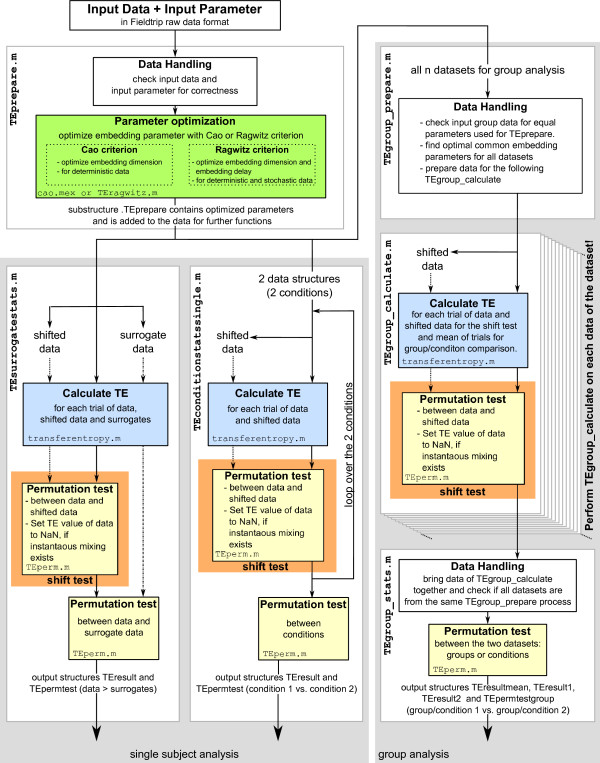
**TRENTOOL workflow**. Structure of main analysis strategies in TRENTOOL. Top left - data preparation; bottom left - comparison to surrogate data; bottom center - comparison of two conditions in one unit of observation; right column - analysis suite for group comparison. Function names for user interaction on the left of each box. Subroutines names at the bottom of shaded areas for parameter estimation (green), TE calculation (blue), shift testing (orange) and general permutation testing (yellow). Arrows indicate the passing of datasets. For details see text.

TRENTOOL provides the core TE estimation routines and algorithms to estimate the necessary parameters from the data - both will be described in detail in the subsection on computational aspects, below. To enable the use of the TE metric in search of directed interactions the metric is embedded in a framework of statistical tests that detect presence and modulations of interactions according to one of three possible analysis strategies (Figure 1):

1. A comparison of TE values from the original data with those of surrogate data in order to detect a directed interaction.

2. A comparison of TE values over trials between two conditions in a single unit of observation (e.g. a single subject) to detect a modulation of directed interaction strength.

3. A comparison of TE values either between two groups of subjects (e.g. patients versus healthy controls) for one condition or between two conditions within a group of subjects, again to detect modulations in the strength of directed interactions.

In the following we describe input data and analysis configuration formats. Then we explain the use of the preparatory function that estimates analysis parameters from the data and that is common to all analyses in TRENTOOL. In this context we also provide details on the set of core functions of TRENTOOL that the user interacts with to follow one of the three analysis strategies above. Last we provide a detailed description of the flow of data in TRENTOOL, aimed at users who want to adapt the toolbox to their own needs. This description (see *Architecture and detailed description*, below) may be safely skipped if the reader is not interested in the architecture of TRENTOOL.

#### Input data and configuration parameters

The input data format is a MATLAB structure containing the fields *trial, time, label*, and *fsample*. The fields *trial *and *time *have to be cell arrays with the length of the number of trials. Each cell of the field *trial *contains the data matrix (number of channels × number of samples) for each trial, and each cell of the field time includes a vector (1 × number of samples) with the time indices (in seconds) for each trial. The cell array *label *stores the channel names (label strings) and *fsample *contains the scalar value of the sampling rate (in Hertz). At the moment this is identical to the FieldTrip raw data format (version 2010-10-25, [28]) and it is planned to keep this compatibility.

Most TRENTOOL functions also require the definition of a set of input *parameters*. These parameters are passed to the function within the fields of a single MATLAB structure typically called *cfg *(for configuration). Some of the parameters in *cfg *have default values which are used in case the field is not defined by the user (see Tables 1, 2, 3, 4).

**Table 1:** The parameters of the function TEprepare.m

**field name of ****cfg.**	default	input	description
sgncmb		strings	Nx2 cell array of specific channel pairs to analyze

channel		strings	cell array of channel names, all combinations will be tested

Path2TSTOOL		string	path to the folder including the required TSTOOL package

toi		vector	first and last time point of the time range of interest (in seconds)

predictiontime_u		integer number	estimated prediction time (in milliseconds)

optimizemethod		string	Method to optimize parameters: 'ragwitz' or 'cao'

ragdim	1 to 10	vector	In case of optimizemethod = 'ragwitz': range of embedding dimensions to scan

ragtaurange		vector	In case of optimizemethod = 'ragwitz': 1 × 2 vector of min and max embedding delays (in units of ACT)

ragtausteps	10	integer number	In case of optimizemethod = 'ragwitz': number of equidistant steps in ragtaurange (minimum 5)

flagNei		string	In case of optimizemethod = 'ragwitz': 'Range' or 'Mass' type of neighbor search

sizeNei		integer number	In case of optimizemethod = 'ragwitz': Radius or mass for the neighbor search according to flagNei

repPred		integer number	In case of optimizemethod = 'ragwitz': repPred represents the number of sample points for which the prediction is performed (it has to be smaller than length(timeSeries) - (d - 1) * tau * ACT - u)

caodim	1 to 10	integer number	In case of optimizemethod = 'cao': indicates the range of embedding dimensions d that is scanned using the Cao criterion to find the optimal dimension

caokth_neighbors	4	integer number	In case of optimizemethod = 'cao': number of neighbors for fixed mass search for cao (controls balance of bias/statistical errors)

tau	1.5	number	In case of optimizemethod = 'cao': embedding delay (in units of ACT)

kth_neighbors	4	integer number	number of neighbors for fixed mass search in TE calculation (controls balance of bias/statistical errors). In case of using optimizemethod = 'cao': kth_neighbors = caokth_neighbors

TheilerT	'ACT'	integer number or 'ACT'	number of temporal neighbors excluded to avoid serial correla-tions in TE calculation (Theiler correction)

trialselect	'ACT'	string	selecting trials: 'no' = use all trials, 'range' = use range of trial numbers, 'ACT' use trials with ACT lower than threshold

actthrvalue		integer number	in case of trialselect='ACT' maximum threshold of the ACT for trial selection

trial_from		integer number	first trial in case of range selection of trials

trial_to		integer number	last trial in case of range selection of trials

maxlag	1000	integer number	the range of lags for computing the ACT: from -MAXLAG to MAXLAG (in samples)

This table contains all possible parameters for the configuration structure cfg of the function TEprepare.m (TRENTOOL Version 1.0)

For integer numbers no type casting has to be performed!

**Table 2:** The parameters for single dataset analysis

**field name of ****cfg.**	default value	input	description
surrogatetype (only in TEsurrogatestats.m)	'trialshuffling'	string	surrogate data for trial(n) will be created by replacing trial n of one channel: 'trialshuffling': trial(n+1) 'trialreverse': reverse of trial(n) 'blockresampling': cuts trial(n) at random point and resamples the trial 'blockreverse1': reverse after blockresampling 'blockreverse2': reverse first block after blockresam-pling 'blockreverse3': reverse second block after blockre-sampling

shifttest	'yes'	string	perform shift test to identify instantaneous mixing between the signal pairs. Values: 'yes' or 'no'

shifttesttype	'TE > TEshift'	string	The shift test can be calculated for the direction TE value of original data greater than the TE values of shifted data (value = 'TE > TEshift') or vice versa (value = 'TEshift > TE'). In this case the alpha level for the shift test is set to 0.1.

shifttype	'predicttime'	string	time shift used in shift test: 'onesample' - shift by one sample into the past; 'predicttime' - shift by the time specified in cfg.predicttime_u in TEprepare.m

permstatstype	'indepsamplesT'	string	'mean' to use the distribution of the mean differences and 'depsamplesT' or 'indepsamplesT' for distribution of the t-values.

numpermutation	190100	integer number	number of permutations in the permutation test

tail	2	integer number	1 or 2 tailed test of significance in the permutation test

alpha	.05	number	significance level for the permutation test

correctm	'FDR'	string	correction method used for correction of the multiple comparison problem - false discovery rate 'FDR' or Bonferroni correction 'BONF'

fileidout		string	the first part of the output filename

dim	optimal embedding dimension found in TEprepare	integer number	number of embedding dimensions; if not specified, the optimal dimension found in TEprepare will be used (recommended option!)

Both single subject analyses functions of TRENTOOL TEsurrogatestats.m and TEconditionstatssingle.m require the same input parameters for the input structure *cfg*. This table contains all possible parameters for the configuration structure cfg of these two functions (TRENTOOL Version 1.0)

For integer numbers no type casting has to be performed!

**Table 3:** The parameters for the group analysis function TEgroup_prepare.m.

**field name of ****cfg.**	default value	input	description
shifttest	'yes'	string	perform shift test to identify instantaneous mixing between the signal pairs. Values: 'yes' or 'no'

shifttesttype	'TE > TEshift'	string	The shift test can be calculated for the direction TE value of original data greater than the TE values of shifted data (value = 'TE > TEshift') or vice versa (value = 'TEshift > TE'). In this case the alpha level for the shift test is set to 0.1

shifttype	'predicttime'	string	time shift used in shift test: 'onesample' - shift by one sample into the past; 'predicttime' -shift by the time specified in predicttime_u in TEprepare.m

dim	optimal embedding dimension found in TEprepare (recom-mended option)	integer number	Number of embedding dimensions. If not specified, the optimal dimension found in TEprepare will be used, which is the recommended option!

tau	(see description)	number	embedding delay in units of ACT If not specified (recommended option), the tau is used as followed: In case of optimizemethod in TEprepare: 'ragwitz' = optimal tau found via ragwitz criterion 'cao' = cfg.tau given by user in TEprepare

This table contains all possible parameters needed for the function TEgroup_prepare.m (TRENTOOL Version 1.0) specified in the input structure *cfg*

For integer numbers no type casting has to be performed!

**Table 4:** The parameters for cfg for the group analysis TEgroup_stats.m.

**field name of ****cfg.**	default value	input	description
design		integer number	matrix containing a row with unit of observation (subject) number and a row with independent variable representing the order of the data input. example for five subjects two conditions: 1234512345 1111122222

uvar		integer number	row in cfg.design which contains the number of the unit of observation (e.g. subject) (in the example: 1)

ivar		integer number	row in cfg.design which contains the independent variable (in the example: 2)

permstatstype	'indepsamplesT'	string	'mean' - use the distribution of the mean differences; 'depsamplesT' (for dependent samples) or 'indepsamplesT' (for independent samples) - use the distribution of the t-values.

numpermutation	190100	integer number	number of permutations in the permutation test

tail	2	integer number	1 or 2 tailed test of significance in the permutation test

alpha	.05	number	significance level for statistical permutation test and correction for multiple comparison

correctm	'FDR'	string	correction method used for correction of the multiple comparison problem - False discovery rate 'FDR' or Bonferroni correction 'BONF'

fileidout		string	the first part of the output filename

This table contains all parameters that can be specified in the input structure *cfg *for the function TEgroup_stats (TRENTOOL Version 1.0)

For integer numbers no type casting has to be performed!

#### Workflow and core functions

As a first step, the input data are prepared for TE analysis using the function TEprepare.m. This function checks the data and the input parameters, selects suitable trials and channels, and optimizes the embedding parameters (for details see section on computational aspects). The results of this function are then added to the input data as a substructure (named *data.*TEprepare). The function TEprepare.m needs input parameters specifying the time range of interest and the channels to be analyzed, the trial selection, the optimization method for the embedding parameters, the parameters associated with that optimization method, and parameters needed for the calculation of TE. Table 1 contains a list of all possible parameters of TEprepare.m, their default values and a more detailed description.

After preparing the data the user can select between three analysis strategies, as explained above:

• For a comparison of TE values from the original data with those of surrogate data, TEsurrogatestats.m creates surrogate data, calculates the TE values, performs a test to detect linear mixing such as volume conduction and performs a permutation test between the TE values of the data and the surrogates. The configuration for this function must specify parameters for these two tests and the method of correcting for multiple comparisons (see table 2 for all parameters, default values and descriptions). In addition, the type of surrogate data has to be specified (see [11] for a discussion of surrogate types for different scenarios).

• For a comparison of TE values over trials between two conditions TEconditionsstatssingle.m is used. This function needs two input datasets to be tested against each other - one for each condition. For both datasets the function TEconditionsstatssingle.m calculates the TE values and performs a shift test. Afterwards this function performs a permutation test between the TE values for the trials of the two datasets.

The configuration parameters for TEconditionstatssingle.m are almost identical to those of TEsurrogatestats.m, above. However, a specification of surrogate data is not necessary.

• The comparison of TE values either between two groups of subjects (e.g. patients versus healthy controls) or between two conditions within a group of subjects makes use of the functions TEgroup_prepare.m, TEgroup_calculate.m, and TEgroup_stats.m. Together, these three connected functions serve to analyze data from one or two *groups *of data. The first function TEgroup_prepare.m checks the input data for a consistent prior usage of TEprepare.m and finds the common optimal embedding parameters for all datasets and prepares the datasets for the function TEgroup_calculate.m. TEgroup_calculate.m calculates the TE values and the shift test for each dataset separately. These computations can be performed by running multiple instances in parallel on different PCs or server nodes at the same time. However, these computations must be started manually (or via a shell script) on all PCs or nodes. The last function - TEgroup_stats.m - checks if the datasets are all from the same TEgroup_prepare.m process and performs the permutation test between the TE values of the two groups of data given as input.

Only two of the three functions in group analysis expect an input configuration - TEgroup_prepare.m and TEgroup_stats. For TEgroup_prepare.m the options of the shift test (see table 3) have to be specified; for TEgroup_stats the assignment of the individual preprocessed input data files to the statistical design (e.g. experimental conditions) and the settings for the statistical test between groups have to be specified (see table 4).

**Figure 2:**
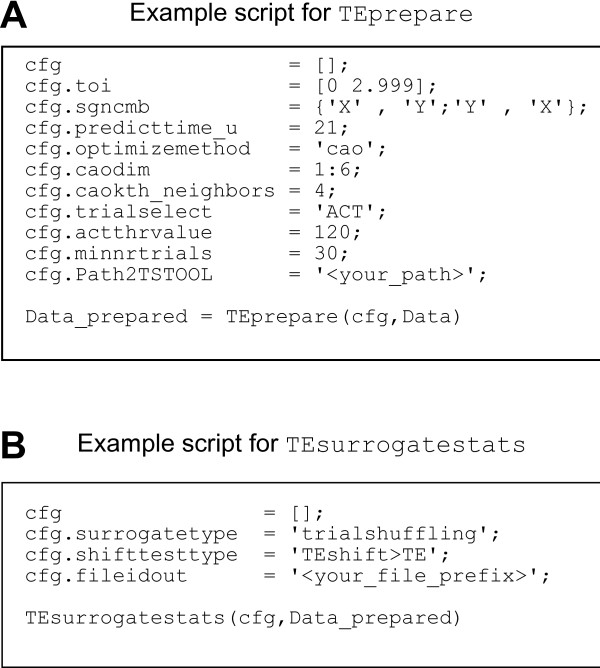
**Example scripts**. Example scripts for using *TEprepare *and *TEsurrogatestats*. A: In the upper script the minimum number of parameters of the configuration structure *cfg *for the use of *TEprepare *are defined. The last line of this script represents the call of the function *TEprepare*. B: The lower script includes the definition of the minimum number of parameters for *TEsurrogatestats *which we used for the analysis of the simulated data.

A typical analysis script is shown in Figure 2. Extensive help on how to call each function and on the possible input parameters is provided by the standard MATLAB help function.

#### Output

The functions TEsurrogatestats.m and TEconditionsstatssingle.m both create two output files: (1) one with the suffix ' *TE output*' containing TE and mutual information (MI) values^3 ^and (2) one with the suffix '_*TEpermtest output*' containing the results of the permutation test. For a group comparison, TEgroup_calculate.m and TEgroup_stats create the corresponding files containing TE/MI, and the statistical output, respectively.

#### Architecture and detailed description

Figure 1 provides a detailed graphical overview of the flow of data in the three analysis strategies, and the corresponding user accessible functions (white boxes) and subroutines (colored areas) : Input data pass through function TEprepare.m (top left box) which checks the data and optimizes the embedding parameters (cao.mex or TEragwitz.m, green shading). With the function TEsurrogatstats.m (box bottom left) it is possible to test single subject data against surrogates. To this end surrogate data are generated, the TE values are calculated (TEvalue.m, blue), shift tests (a combination of TEvalue.m and TEperm.m with special input configuration, orange) are performed to find volume conduction and at the end the data and the surrogates are compared with a permutation test (TEperm.m yellow). To test two conditions against each other in a single subject, the function TEconditionstatssingle.m (bottom center box) computes the TE values (TEvalue.m, blue) and the shift tests (orange) separately for both input datasets, and then the TE values of the two datasets are compared with a permutation test (TEperm.m yellow).

The three functions TEgroup_prepare.m, TEgroup_calculate.m, and TEgroup_stats.m (left box) are used for group analyses. The first function TEgroup_prepare.m, checks the input data for uniform usage of TEprepare.m, finds the common embedding parameters for all datasets and prepares the datasets for passing to the following functions. The next function TEgroup_calculate.m calculates TE values (TEvalue.m, blue) and performs the shift test (orange) for each dataset separately. All the datasets from TEgroup_calculate.m serve then as input to TEgroup_stats.m; this function checks if the datasets are all from the same TEgroup_prepare.m process and performs the permutation test (TEperm.m yellow).

### Definition and computational aspects of transfer entropy

After explaining the use of TRENTOOL and the different possible analysis strategies we will now describe in detail how TE is defined, and how TE estimation, the necessary parameter identification steps, and the statistical testing are implemented in TRENTOOL.

Transfer entropy indicates the presence of directed interactions by measuring predictive, directed information flow from a source *X *to a target *Y *[29], i.e. it quantifies how much the past of a process *X *conditions the transition probabilities of another process *Y*. Thus, - assuming that the two associated time series *X *= *x*_*t *_and *Y *= *y*_*t *_can be approximated by Markov processes - we are interested in the deviation from the following generalized Markov condition:

(1) $$p\left(y_{t+1}|y_{t}^{n},x_{t}^{m}\right)=p\left(y_{t+1}|y_{t}^{n}\right),$$

where $x_{t}^{m}=\left(x_{t},...,x_{t-m+1}\right),y_{t}^{n}=\left(y_{t},...,y_{t-n+1}\right)$, while *m *and *n *are the orders (memory) of the Markov processes *X *and *Y*, respectively. When the transition probabilities or dynamics of *Y *are independent of the past of *X*, Eq. 1 is fully satisfied, and we infer an absence of directed interaction from *X *to *Y*. To measure the departure from this condition (i.e. the presence of directed interactions), Schreiber [12] used the expected Kullback-Leibler divergence between the two probability distributions at each side of Eq. 1 and defined the TE from *X *to *Y *as

(2) TE(X→Y)=∑yt+1,ytn,xtmp(yt+1,ytn,xtm) log(p(yt+1,ytn,xtmp(yt+1|ytn)).

**Figure 3:**
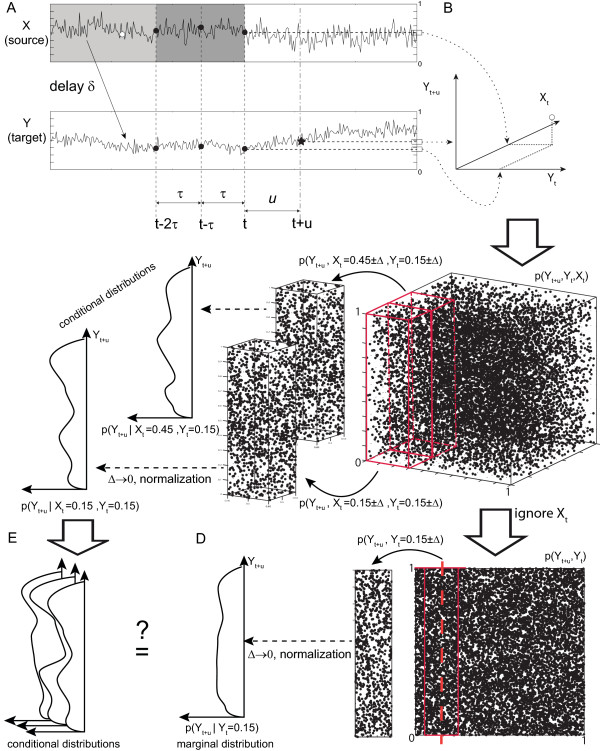
**Graphical visualization of TE**. (A) Coupled systems *X *→ *Y*. To test directed interaction *X *→ *Y *we predict a future *Y*(*t *+ *u*) (star) once from past values (circles) of *Y *alone: $Y_{est}^{\left(Y\right)}\left(t+u\right)=F\left(Y\left(t\right),Y\left(t-\tau \right),Y\left(t-2\tau \right)\right)$, once from past values of Y and X: $Y_{est}^{\left(X,Y\right)}\left(t+u\right)=F\left(Y\left(t-\tau \right),Y\left(t-2\tau \right),X\left(t-\tau \right),X\left(t-2\tau \right)\right)$. *d *- embedding dimension, *τ *- embedding lag. (B) Embedding. *Y*(*t *+ *u*), *Y*(*t*), *X*(*t*) - coordinates in the embedding space, repetition of embedding for all *t *gives an estimate of the probability *p*(*Y*(*t *+ *u*), *Y*(*t*),*X*(*t*)) (part C, embedding dimensions limited to 1).(C) *p*(*Y*(*t *+ *u*)|*Y*(*t*),*X*(*t*)) - probability to observe *Y*(*t*+*u*) after *Y*(*t*) and *X*(*t*) were observed. This probability can be used for a prediction of the future of *Y *from the past of *X *and *Y*. Here, *p*(*Y*(*t *+ *u*)|*Y*(*t*), *X*(*t*)) is obtained by a binning approach. We compute *p*(*Y*(*t *+ *u*) ± Δ, *Y*(*t*) ± Δ,*X*(*t*) ± Δ), let Δ → 0 and normalize by *p*(*Y*(*t*),*X*(*t*))). TRENTOOL computes these densities via a Kernel-estimator. (D) *p*(*Y*(*t *+ *u*)|*Y*(i)) predicts *Y*(*t *+ *u*) from *Y*(*t*), without knowing about *X*(*t*). It predicts the future of *Y *from the past of *Y *alone. (E) If the past of *X *is irrelevant for prediction, the conditional distributions *p*(*Y*(*t *+ *u*)|*Y*(*t*), *X*(*t*)), should be all equal to *p*(*Y*(*t *+ *u*)|*Y*(*t*)). Differences indicate directed interaction from *X *to *Y*. Their weighted sum is transfer entropy.

For a graphical representation of the quantities involved please refer to Figure 3. Note that under very general conditions transfer entropy is equivalent to a conditional mutual information formulation independently introduced by Paluš [30].

Note that in the original formulation a prediction is generated for one sample ahead. As interactions in general may have long interaction delays well above the time covered by $x_{t}^{m}=\left(x_{t},...,x_{t-m+1}\right),y_{t}^{n}=\left(y_{t},...,y_{t-n+1}\right)$, we generalized the above definition of TE for two observed time series *x*_*t *_and *y*_*t *_by including a general prediction time *u*:

(3) TE(X→Y)=∑yt+u,ytdy,xtdxpyt+u,ytdy,xtdx logpyt+u|ytdy,xtdxpyt+u|ytdy,

where *t *is a discrete valued time-index and *u *denotes the prediction time, a discrete valued time-interval. $y_{t}^{d_{y}}$ and $x_{t}^{d_{x}}$ are *d*_*x*_- and *d*_*y*_-dimensional delay vectors as detailed in the next section.

Transfer entropy naturally incorporates directional and dynamical information, because it is inherently asymmetric and based on transition probabilities. Transfer entropy is only well defined if all the marginal and joint probability distributions are non-singular, e.g. not delta-distributed. This excludes situations where time series are related by fully deterministic functions, i.e when one-to-one mapping exists between the states of the two systems. No causal relation can be inferred in those cases and this is reflected by a breakdown of the definition of TE.

#### Computation of transfer entropy

In this subsection we detail how to obtain a data-efficient estimation of equation 3 from the raw signals.

Prior to estimating TE it is necessary to reconstruct the state space of the raw data. In this work, we use Takens' delay embedding [31] to map our scalar time series into trajectories in a state space of possibly high dimension. The mapping uses delay-coordinates to create a set of vectors or points in a higher dimensional space according to

(4) $$\begin{array}{l} x_{t}^{d}=(x(t),x(t-\tau ),x(t-2\tau ),...,\\ \;\;\;x(t-(d-1)\tau )). \end{array}$$

This procedure depends on two parameters, the dimension *d *and the delay *τ *of the embedding. For deterministic systems and data of infinite length all choices of *τ *are equivalent and the correct dimension *d *can be estimated. For real data containing stochastic driving forces and noise, only approximate algorithms for the determination of *d *and *τ *exist. For a causality analysis according to Wiener's principle, however, it is not necessary to recover the true dynamics of the systems under investigation (their 'attractors'), but to obtain an optimal prediction of the future of each signal from its past, so that the prediction to be improved upon is not artificially imprecise^4^. With this in mind we may use approximate criteria to determine *d *and *τ*, as they have been proposed by Cao [32] and Ragwitz and Schreiber [12]. In Cao's criterion *τ *is chosen ad hoc - a popular option is to take the embedding delay *τ *as the first zero of autocorrelation function of the signal or the first minimum (if any) of the auto-information - and *d *is determined based on a false neighbor criterion; in Ragwitz' criterion *d *and *τ *are jointly optimized to minimize the prediction error of a local predictor. Both algorithms are described in more detail below.

After having reconstructed the state spaces of any pair of time series, we are now in a position to estimate the TE between their underlying systems. We proceed by first rewriting Eq. 3 as sum of four Shannon entropies according to

(5) $$\begin{array}{llll} TE\left(X\to Y\right)= & S\left(y_{t}^{d_{y}},x_{t}^{d_{x}}\right)-S\left(y_{t+u},y_{t}^{d_{y}},x_{t}^{d_{x}}\right)\; & & \;\\ & \;\;+S\left(y_{t+u},y_{t}^{d_{y}}\right)-S\left(y_{t}^{d_{y}}\right).\; & & \;\\ & \; & \end{array}$$

Thus, the problem amounts to computing this combination of different joint and marginal differential entropies. Here, we used a data efficient approach to compute TE that is based on nearest neighbors techniques and the Kraskov-Stögbauer-Grassberger estimator, and is a variation of the approaches described in (Gomez-Herrero G, Vicente R, Wu W, Pipa G, Egiazarian K: Assessing causal relationships from an ensemble of multivariate time series, submitted. and [11,14]).

Nearest-neighbor techniques estimate Shannon differential entropies from the statistics of distances between neighboring data points once embedded in a given space. They have the advantage of being as local as possible given the available data, and to offer a good data-efficiency, which is necessary to estimate entropies in high-dimensional spaces from limited real data [15,33]. The assumption behind nearest-neighbor estimators is only a certain smoothness of the underlying probability distribution. Nearest-neighbor estimators can therefore be considered as non-parametric techniques.

Unfortunately, it is problematic to estimate TE by simply applying a nearest-neighbor estimator (e.g. Kozachenko-Leonenko estimator) separately to each of the terms appearing in Eq. 5. The reason is that the dimensionality of the spaces involved Eq. 5 can differ largely across terms. Thus, fixing a given number of neighbors for the search will set very different spatial scales (range of distances) for each term. Since the error bias of each term is dependent on these scales, the errors would not cancel each other but accumulate. We therefore used the Kraskov-Grassberger-Stögbauer estimator which handles this problem by only fixing the number of neighbors in the highest dimensional space and by projecting the resulting distances to the lower dimensional spaces as the range to look for neighbors there [14]. After adapting this technique to the TE formula (Gomez-Herrero G, Vicente R, Wu W, Pipa G, Egiazarian K: Assessing causal relationships from an ensemble of multivariate time series, submitted.), the estimator we use can be written as

(6) $$\begin{array}{c} TE\left(X\to Y\right)=\psi \left(k\right)+\langle \psi \left(n_{y_{t}^{d_{y}}}+1\right))\\ -\psi \left(n_{y_{t+u}y_{t}^{d_{y}}}+1\right)\\ {(-\psi \left(n_{y_{t}}^{d_{y}}x_{t}^{d_{x}}\right)\rangle }_{t}, \end{array}$$

where the distances to the *k*-th nearest neighbor in the highest dimensional space (spanned by $y_{t+u},y_{t}^{d_{y}},x_{t}^{d_{x}}$) define the radius of the spheres for the counting of points *n*_*Z *_in all the marginal spaces *Z *involved. *ψ *denotes the digamma function, while the angle brackets (〈·〉*t*) indicate an averaging over different time points.

The use of equation 6 implies that the state spaces of the signals have been reconstructed. Choosing a value for the embedding dimension *d *is a crucial decision in this respect that is far from trivial. For instance, if the value of *d *is chosen too low, it can be insufficient to unfold the state space of a system leading to incorrect results in the neighbor search and consequently degrade the meaning of any TE measure. On the other hand, when using an embedding dimension which is higher than necessary, samples in the high dimensional space get too sparse to estimate the probability density correctly. This will make the estimates less accurate and enlarges the computation time.

Two different optimization algorithms to find the optimal embedding dimension for the data are available in TRENTOOL. For deterministic (chaotic) systems the Cao criterion offers an algorithm based on the computation of false neighbors [32]. For stochastically driven systems the Ragwitz criterion provides parameters that allow for an optimal prediction of future states [34]. Both optimization criteria are explained in more detail in the next two paragraphs.

##### Cao criterion

The Cao criterion described in [32] is a method to determine the minimum embedding dimension of deterministic time series by analyzing neighborhood relations in various dimensions. In the Cao criterion the relative increase in distance between nearest neighbors in *d*-dimensions that is brought about by incrementing the dimension *d *by 1 is defined as

(7) $$a\left(t,d\right)=\frac{\left||x_{t}^{d+1}-x_{t^{\prime }\left(t,d\right)}^{d+1}|\right|}{\left||x_{t}^{d}-x_{t^{\prime }\left(t,d\right)}^{d}|\right|}.$$

where *t *= 1, 2,..., *N - dτ *and ||·|| is some measure of Euclidean distance in *d *and *d *+ 1 dimensions. The vector $x_{t}^{d}$ and its nearest neighbor $x_{t^{\prime }\left(t,d\right)}^{d}$ are nearest neighbors in the *d*-dimensional space. Their distance is also evaluated in *d *+ 1 dimensions in the numerator of the formula (7).

A neighbor is called a true neighbor if two points are close both in the *d*-dimensional reconstructed space and in the (*d *+ 1)-dimensional reconstructed space. Otherwise, these neighbors are called false neighbors. For an optimal embedding we would like to increase the embedding dimension *d *just up to the point where no false neighbors are found. Unfortunately, the classification into true and false neighbors depends on choosing a threshold value for *a*(*t*, *d*) and it is impossible to define a threshold value that works independent of the dimension *d *and of the time points *t*. Hence, Cao [32] proposed to use the following quantity to define the minimum embedding dimension:

(8) $$E\left(d\right)=\frac{1}{N-d\tau }\overset{N-d\tau }{\underset{t=1}{\sum }}a\left(t,d\right).$$

*E*(*d*) is the mean value of all *N *instances of *a*(*t, d*) and is dependent only on the dimension *d *and the time lag *τ*. The variation *E*1(*d*) from *d *to *d *+ 1 is defined as

(9) $$E1\left(d\right)=\frac{E\left(d+1\right)}{E\left(d\right)}.$$

With increasing *d E*1(*d*) stops changing at some point [32]. The *d *of this transition is used as embedding dimension and therefore the Cao criterion is only dependent on the embedding delay *τ *as a free parameter. A popular *ad hoc *choice for *τ *is the first zero of the autocorrelation function or the first minimum (if any) of the auto-information.

In TEprepare.m the optimal embedding dimension is found by minimizing

(10) E1(d-1)+E1(d+1)-2*E1(d)

This optimal embedding dimension *d *from the Cao criterion and the *τ *which was defined in advance are then used for the calculation of the TE values in the downstream functions of the toolbox.

##### Ragwitz criterion

In most of the cases real data and especially neuroscience data are not purely deterministic - as it is implied in Cao's algorithm. Hence, in TRENTOOL we implemented a method which optimizes the embedding dimension *d *and the embedding delay *τ *for deterministic and stochastic data from Markovian processes. Optimality of the embedding here refers to a minimal prediction error for future samples of the time series. The Ragwitz criterion, described in [34], predicts a future value ofthe signal based on estimates of the probability densities of future values of its nearest neighbors after embedding. The actual prediction is based on some suitable parameter of the estimated probability distributions, e.g. their mean. This case corresponds to a minimization of the mean squared prediction error and results in the local constant predictor [35,36]. In TRENTOOL we aim to minimize exactly this mean squared prediction error - as this is implicitly required by Wiener's principle. Hence, we use the local constant predictor, where an estimate of the unobserved future *x*_*t+u *_of the signal $x_{t}^{d_{x}}$ is obtained from the mean of the futures *x*_*t'+u *_of its neighbors $x_{t^{\prime }}^{d_{x}}$:

(11) $${\hat{x}}_{t+u}=\frac{1}{\left|U_{t^{\prime }}\right|}\underset{U_{t^{\prime }}}{\sum }x_{t^{\prime }+u}\;,$$

where *U*_*t' *_is the set of vectors $x_{t^{\prime }}^{d_{x}}$ which are within a volume with radius e around $x_{t}^{d_{x}}$:

(12) $$U_{t^{\prime }}=\left\{x_{t^{\prime }}^{d_{x}}:||x_{t^{\prime }}^{d_{x}}-x_{t}^{d_{x}}||\leq \varepsilon \right\}$$

We then optimize parameters for *d *and *τ *such that we minimize the mean squared prediction error:

(13) $$e^{2}=\underset{t}{\sum }{\left(x_{t+u}-{\hat{x}}_{t+u}\right)}^{2}$$

Using the Ragwitz criterion in TRENTOOL means to scan different embedding dimensions *d *and embedding delays *τ *which are given as parameters of the input configuration.

#### Statistical Testing

Information theoretic estimators often come with a bias for finite datasets (see e.g. [37]) and TE is no exception [38]. Therefore, absolute TE values have limited meaning and TRENTOOL uses TE only as a *metric *in a statistical test of the null hypothesis of independence. As the distribution of the test statistic under the null hypothesis is unknown, these tests have to be performed non-parametrically, e.g. via permutation testing (see e.g. [39]).

##### Permutation Testing

A permutation test is a non-parametrical statistical significance test, which is used to test whether two (or more) groups ofdata are *exchangeable *[39]. The basic approach is the following: Decide on a metric to measure the effect in question (e.g. raw TE values, t-statistics, differences of means, etc). Then calculate that test statistic on the data (t_obt_). Afterwards pool the data and repeat the following *n *times: shuffle the data, split the data in two (or more) groups, calculate the test statistic t_i* _for the reshuffled data. This gives the distribution of the test statistic under the null hypothesis of exchangeability. The null hypothesis can now be rejected or retained by comparing the actual test statistic t_obt _to this distribution of t_i*_.

The main advantages of permutation tests are that they exist for any test statistic, regardless of whether its distribution is known or not.

In TRENTOOL permutation tests are implemented in the internal function TEperm.m for the statistical comparison in three different contexts:

1. for a comparison of data with a shifted version of the data to find instantaneous mixing in the data (e.g. volume conduction, shared noise) - this procedure is called shift-testing and explained below -,

2. for a comparison of the data with surrogate data,

3. and for a comparison of (groups of) datasets to find significant differences in TE between them.

##### Shift-Testing

Real data typically contain not only the signal of interest but are contaminated by noise. Moreover, this noise contribution may not be independent for two signals that are evaluated for directed interactions using TE. Typical examples are shared 50 Hz signals, the effect of volume conduction in Electroencephalography and field spread effects in Magnetoencephalography. Shared noise with a non- zero autocorrelation can have various effects on measures based on Wiener's definition of causality. As a general rule, false positive detection of a causal interaction from the less noisy to the noisier signal is likely to occur [11,40]. In order to avoid false positive detection of a causal interaction due to instantaneously shared noise we devised a so called shift test [11,27]. In this test, TE from a signal *X*(*t*) to a signal *Y*(*t*) with a prediction time *u *is computed twice - once for the original signals and once by replacing *X*(*t*) by a time-shifted version *X*'(*t*) = *X*(*t *+ *u*). The effect of this time shift on *X *is that samples now occur *u *time steps earlier in the shifted signal than in the original one. Since we expect a time delay *δ *> 0 for the coupling from *X *to *Y*, the new set of values for *X' *cannot be causally related to *Y *(given a correct choice for the prediction time *u *approximately equal to the interaction delay *(δ) *between the signals, and given no instantaneous mixing). Hence, if we were dealing with a truly causal interaction, we effectively loose useful samples that served to predict the future of *Y *and replace them by acausal ones. Therefore TE values should drop, i.e. *TE*(*X *→ *Y*) >*TE*(*X' *→ *Y*). In contrast, if we observed a causal interaction because of an instantaneous common noise contribution, this noise signal now appears *u *samples earlier in the shifted signal *X'*, and allows perfectly to predict its own appearance in *Y u *samples later. In this case, we will see an increase in TE values, indicating instantaneously shared signal or noise.

In TRENTOOL we formalized this argument in the following way: For each trial *i *we compute both, *TE*_*i*_(*X *→ *Y*) and *TE*_*i*_(*X' *→ *Y*). Then we compare the two distributions of TE values for the original and the shifted signal pair by means of a permutation test. If TE values for the shifted signal pair are significantly larger than for the original one then we discard the hypothesis that there is a directed interaction^5^. Note, that this result should not be interpreted as the proof of absence of directed interaction but rather means that under these circumstances we cannot make any statement about a true interaction due to the presence of instantaneously shared noise.

## Results

### Validation for simulated data

We tested our implementation of Transfer Entropy with a representative set of simulated data which mimic electrophysiological recordings and where we have control over all parameters such as coupling direction, delay and strength^6^.

For each simulation, 100 datasets were generated with 40 trials, each 3000 samples long. All signals *X *and *Y*, or *X*_ε _and *Y*_ε _in case of linear mixing, were evaluated with first TEprepare and then with TEsurrogatestats using the default parameters for the functions as listed in table 1 and 2, and using the free parameters exactly as shown in the example scripts in Figure 2 (A) for TEprepare and (B) for TEsurrogatestats if not specified otherwise. The following paragraphs we describe motivation, simulation setup and results.

#### Sensitivity analysis - impact of embedding parameters

The sensitivity of the TE metric mostly depends on two parameters - the prediction time *u *that quantifies the expected interaction delay between the two systems and which has to be set by the user and the combination of embedding dimension *d *and delay *τ*, which is estimated by either the Cao (only *d*) or the Ragwitz criterion (*d *and τ). The following two simulations demonstrate the impact of *u *and *d *on sensitivity.

##### Impact of correct prediction time *u*

To investigate the influence of the choice of the prediction time *u *on TE analysis results, we simulated two unidirectionally quadratically coupled autoregressive processes with order

10 (AR(10))

(14) $$X\left(t+1\right)=\overset{9}{\underset{i-0}{\sum }}\alpha_{i}X\left(t-i\right)+0.1\eta_{x}\left(t\right)$$

(15) $$\begin{array}{llll} Y\left(t+1\right)= & \overset{9}{\underset{i=0}{\sum }}\alpha_{i}Y\left(t-i\right)+0.1\eta_{y}\left(t\right)\; & & \;\\ & +\gamma X{\left(t+1-\delta \right)}^{2}\; & & \;\\ & \; & \end{array}$$

where all *η *are Gaussian white noise processes with unit variance, the coupling constant *γ *was chosen such that the coupling term contributes 50% of the variance of the final *source *signal Y, and *δ *is the coupling delay and was set to 21 samples. For the evaluation of this dataset, we scanned *u *from 1 to 40 samples.

##### Results

**Figure 4:**
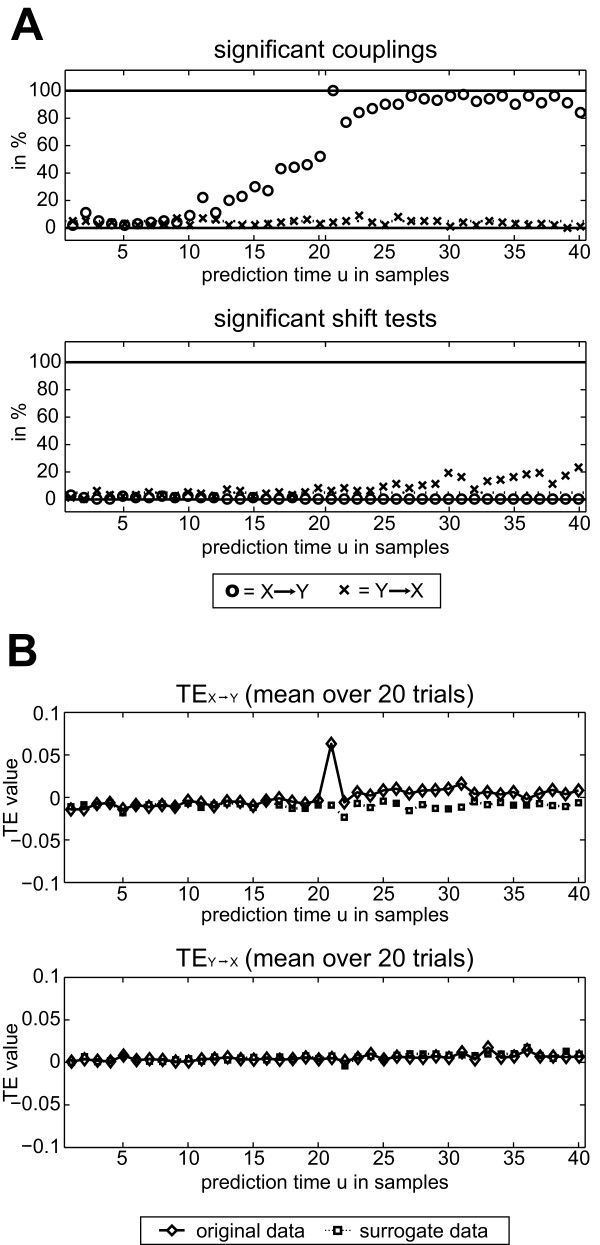
**Influence of prediction time on TE**. Results for quadratically coupled AR(10) processes with a coupling delay *δ *of 21 samples (*X *to *Y*). (A) Upper panel - Detection rates versus prediction time u. Open circles: detection of coupling from *X *to *Y *(correct detections); Cross: detection of coupling from *Y *to *X *(false positives). Interactions can be detected best, if the prediction time *u *is set to the coupling delay (*u *= *δ *= 21). Lower panel: Shift test detection rates (B) TE values versus prediction time *u. *Solid line: TE values of the generated data. Dotted line: TE values for trial-shuffled surrogate data. The difference between the TE values of the data and the surrogates is largest when the prediction time *u *is set to the coupling delay.

The rate of correct detections of an interaction peaked at *u *= 21, which is equal to the simulated coupling delay (Figure 4A). At this optimal prediction time of *u *= 21 we also found the highest TE values (Figure 4B). This result held irrespective of the coupling type simulated (linear, quadratic, threshold; data not shown). Beyond this peak, detection rates first dropped with increasing *u *and then showed a second broad peak, however, without reaching the maximal level again. This latter result was specific to the data analyzed.

At the optimal prediction time *u *= 21, *X *→ *Y *was detected for all 100 datasets. The mean p-value over all 100 datasets for *u *= 21 was: *X *→ *Y *= 0.0000050; *Y *→ *X *= 0.1316. This mean p-value at *u *= 21 for *X *→ *Y *was significant after a post hoc Bonferroni correction for the multiple prediction times scanned.

The shift test was applied to detect instantaneous mixing. As no instantaneous mixing was implemented in this dataset, results here serve to evaluate its false positive rate. As expected, the shift test did not detect instantaneous mixing above chance level (0.1) for any *u *(Figure 4A, lower panel) for the analysis of *X *→ *Y*. For the reverse direction, *Y *→ *X *we observed a detection rate of instantaneous mixing slightly higher than chance level. This was expected because for certain combinations of *δ *and *large *prediction times *u *-the shifting effectively reverses the coupling delay and thus increases TE for the shifted data compared to the original ones. This does not decrease overall sensitivity, however, as no coupling in this direction would have been observed anyway.

The corresponding raw TE values are plotted in Figure 4B. The maximum value was obtained for *X *→ *Y *at *u *= 21 which is in agreement with equation 15 and the results above.

##### Impact of optimal embedding dimension *d*

To investigate the influence of the embedding dimension *d *we used the same kind of simulated data as in the preceding paragraph. Here, we scanned *d *from 3 to 8, and additionally added a varying amount of noise to the data from 20% to 200% in steps of 20% of the original variance of the data.

##### Results

**Figure 5:**
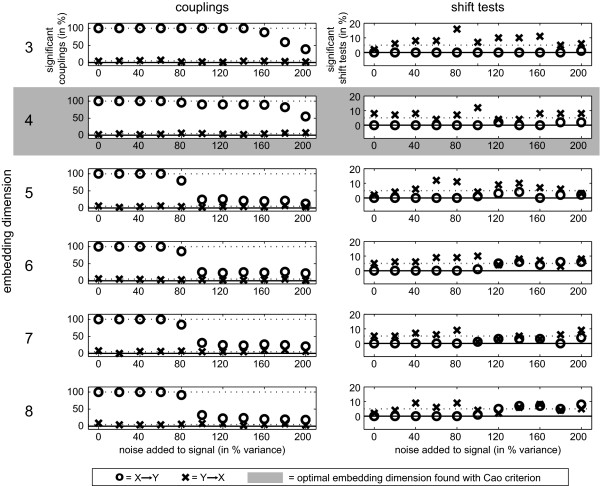
**Influence of embedding dimension on TE**. Results for coupled AR(10) processes with a coupling delay of 21 samples (*X *to *Y*). Left: Results of permutation testing for varying noise added to the signals *X *and *Y *(20-200 in % variance of the noise free signal) for 100 datasets each. Embedding dimensions from 3 to 8 (top to bottom). The embedding dimension that was found by the Cao criterion is highlighted in gray. Open circles: detection of coupling from *X *to *Y *(correct detections); Cross: detection of coupling from *Y *to *X *(false positives). Right: Results of the corresponding shift tests Solid lines represent 0 and the dotted lines the values 5% and 100%.

For all noise levels, the optimal embedding dimension was estimated by the Cao criterion to be *d *= 4 (Figure 5). For all tested embedding dimensions, the coupling *X *→ *Y *was detected robustly for noise levels smaller than 80% of the variance of the original signals (Figure 5, left panel). At higher noise levels, the detection rate decreased for dimensions larger than the optimal embedding dimension obtained from Cao's criterion (4).The mean p-values (permutation test, FDR *q *< 0.05) for the optimal embedding dimension 4 over all 100 datasets and noise levels were *X *→ *Y *= 0.005, and *Y *→ *X *= 0.302. The shift test was at or below chance level for almost all *d *for the direction *X *→ *Y*, as desired. For the reverse direction *Y *→ *X *the rates of positive shift tests were at chance level on average but exhibited some fluctuations.

#### Specificity analysis

While we are interested in a measure that is sensitive enough to detect directed interactions, we must be concerned with its robustness, i.e. we want to have a measure that delivers false positive results at a specified rate only. The measure should exhibit this low false positive rate even under unfavorable conditions. Common examples of such unfavorable conditions are shared noise, e.g. due to line noise, and instantaneous linear signal mixing as it arises due to volume conduction or field spread in EEG or MEG, respectively.

##### Robustness against linear mixing

This part of the study was aimed at demonstrating the applicability of TE analysis in case of linear signal mixing. For these cases we demonstrate the robustness against false positive detection of directed interactions that is provided by the shift-test described above. To this end, we simulated five test cases in the following way:

(A) As an example of no interaction, two uncoupled Gaussian white noise processes *X*, *Y *were generated.

(16) $$X\left(t\right)=\eta_{x}\left(t\right)$$

(17) $$Y\left(t\right)=\eta_{y}\left(t\right)$$

where *η*_*x *_and *η*_*y *_are Gaussian white noise processes with unit variance.

(B) To simulate volume conduction effects, one Gaussian white noise process *Z*(*t*) was generated and mixed onto two noisy sensors *X*_ε _and *Y*_ε_.

(18) $$Z\left(t\right)=\eta_{x}\left(t\right)$$

(19) $$X_{\varepsilon }\left(t\right)=\varepsilon Z\left(t\right)+\eta_{sx}\left(t\right)$$

(20) $$Y_{\varepsilon }\left(t\right)=\left(1-\varepsilon \right)Z\left(t\right)+\eta_{sy}\left(t\right)$$

with ε ∈ {0.05 0.1 0.2 0.3 0.4 0.5} and where *η*_*x *_is Gaussian white noise of unit variance representing the innovation of the AR process. *η*_*sx *_and *η*_*sy *_are Gaussian white noise sources that contribute 25% variance to the final signals and represent sensor (observation) noise. The mixing is parametrized by ε with ε = 0.5 leading to identical signals apart from sensor noise.

(C) To investigate linear mixing in a two-source scenario without coupling, two independent Gaussian white noise processes *X, Y *were generated as in case (A) and linearly mixed similar to case (B):

(21) $$X\left(t\right)=\eta_{x}\left(t\right)$$

(22) $$Y\left(t\right)=\eta_{y}\left(t\right)$$

(23) $$X_{\varepsilon }\left(t\right)=\left(1-\varepsilon \right)X\left(t\right)+\varepsilon Y\left(t\right)+\eta_{sx}\left(t\right)$$

(24) $$X_{\varepsilon }\left(t\right)=\left(1-\varepsilon \right)X\left(t\right)+\varepsilon Y\left(t\right)+\eta_{sy}\left(t\right)$$

with ε ∈ {0.05 0.1 0.2 0.3 0.4 0.5} and where *η*_*sx *_and *η*_*sy *_are Gaussian white noise sources representing sensor noise that contribute 25% variance to the final signals. A mixing parameter ࿐ of 0.5 results in identical signals apart from the noise differences.

(D) To investigate the interplay between linear mixing and true coupling, two stable AR(10) processes with unidirectional quadratic coupling were mixed onto two noisy channels via the same symmetric linear mixing system as in case (C).

(25) $$X\left(t+1\right)=\overset{9}{\underset{i=0}{\sum }}\alpha_{i}x\left(t-i\right)+0.1\eta_{x}\left(t\right)$$

(26) $$Y\left(t+1\right)=\overset{9}{\underset{i=0}{\sum }}\alpha_{i}y\left(t-i\right)+\gamma X{\left(t+1-\delta \right)}^{2}+0.1\eta_{y}\left(t\right)$$

(27) $$X_{\varepsilon }\left(t\right)=\left(1-\varepsilon \right)X\left(t\right)+\varepsilon Y\left(t\right)+\eta_{sx}\left(t\right)$$

(28) $$Y_{\varepsilon }\left(t\right)=\left(1-\varepsilon \right)Y\left(t\right)+\varepsilon X\left(t\right)+\eta_{sy}\left(t\right)$$

Where ε ∈ {0.05 0.1 0.2 0.3 0.4 0.5}. All *η *are Gaussian white noise processes, the coupling constant γ was chosen such that the coupling term contributes 50% of the variance of the final signal *Y *before adding sensor noise. *δ *represents the coupling delay and was set to 21 samples. This test case has a true delayed coupling and also different levels of linear mixing ε. It serves to investigate up to which level of linear mixing the delayed coupling can still be detected.

(E) - Influence of 50 Hz noise. To investigate the influence of 50 Hz noise on TE analysis, we generated two unidirectionally quadratically coupled AR(10) processes in the same way as in the previous case (D) but without the linear mixing. To the original data *X*(*t*) and *Y*(*t*) we (1) added 50 Hz noise and (2) filtered the data with a 4th order two-pass Butterworth IIR bandstop filter for 49-51 Hz, to also simulate both the effect of line noise contamination as well as the effect of filtering for line noise removal. TE analyses were performed for all three data sets, original, 50 Hz noise added, and filtered.

##### Results

**Figure 6:**
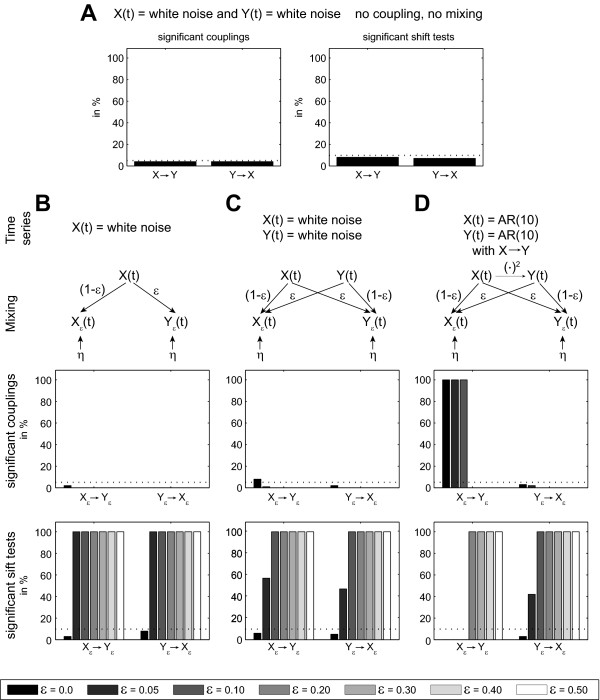
**Robustness of TE analysis against common noise and volume conduction**. (A) Independent white Gaussian noise processes *X*,*Y*. Left: Significant couplings (i.e. false positives) at a test level of 5%. Right: significant shift test results at a test level of 5%. (B) Single Gaussian white noise process *X *observed by two noisy sensors *X*_ε_, *Y*_ε_. (C) Two independent Gaussian white noise sources *X*,*Y*, observed via a linear mixing system on two noisy sensors *X*_ε_, *Y*_ε_. (D) Mixing as in C. Here, the two source AR(10) processes are coupled by a quadratic coupling function.

In the first case (A) of two independent Gaussian white noise processes the detection rate of directed interactions and of volume conduction was at chance level (Figure 6 A).

In the second case (B) of only one Gaussian white noise process mixed onto two noisy sensors, no directed interactions were present. In this case, coupling was detected at rates at or below chance level for all ε (Figure 6 B). For ε = 0 the shift test was expected to be non-significant, because no instantaneous mixing was present. For ε > 0, the shift test did robustly detect the instantaneous mixing in 100% of simulated cases.

In the third case (C) of two *independent *Gaussian white noise processes mixed onto two noisy sensors, directed interactions were found at chance level for ε = 0. For larger e directed interactions were not found at all (Figure 6C). This is because TRENTOOL eliminates positive results when the shift returns a positive result (see below). In both directions (*X *→ *Y *and *Y *→ *X*) instantaneous mixing was not detected by the shift test for ε = 0.0 - which is the correct result. For weak instantaneous mixing (ε = 0.05), the detection rate was about 50%. For stronger mixing (ε > 0.1) the volume conduction was detected robustly.

**Figure 7:**
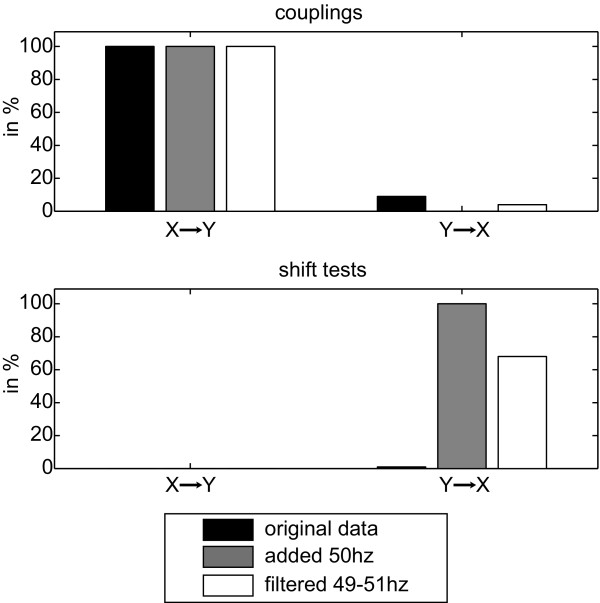
**Influence of 50 Hz noise**. Influence of 50 Hz noise on TE analysis. Results of permutation testing (top) and the appropriate shift tests bottom for the original dataset (black), after adding 50 Hz noise to the data (gray), and after filtering out 49-51 Hz (white) of 100 datasets each. (Left) Analysis *X *→ *Y*, the direction in which coupling was present; (Right) Analysis *Y *→ *X*.

In the fourth case (D) of two unidirectionally quadratically coupled AR(10) processes that where mixed onto two noisy sensors the directed interaction was detected for small instantaneous mixing levels ε < 0.3, while instantaneous mixing was found instead for the larger mixing levels (Figure 6D). Note, that either instantaneous mixing or directed interactions can be detected by construction of the algorithm. The output of the algorithm thus indicates whether the influence of interaction or mixing dominates the dependencies between signals. As in the third case (compare Figure 6 C), the instantaneous mixing for *Y *→ *X *was detected robustly for ε > 0.05. For the fourth case (E), the detection of the directed interaction *X *→ *Y *was neither impaired by the 50 Hz noise nor by the filtering (Figure 7). The opposite direction *Y *→ *X *did show false positives only at chance rate (Figure 7). The shift test was not significant in the direction of coupling *X *→ *Y*, but did robustly detect instantaneous mixing for the opposite direction *Y *→ *X *if 50 Hz was present and also at a rate of 68% when the signal had been filtered, indicating that filtering does not remove all effects of the common noise.

##### Application to group data

To demonstrate the use of TRENTOOL for the analysis of group data, we simulated data sets for 15 subjects and two conditions. Each of the 30 datasets contained 4 simulated channels with 40 trials, each 3000 samples long. We assigned the simulated channels to four channels labeled *F3, F4, T7 *and *T8*. All channels contained AR(10) time series as described in equation 25. The first conditions had a unidirectional quadratic coupling from *F3 *to *T8 *as described in equation 26, whereas the second condition was implemented with a unidirectional quadratic coupling from *T7 *to *F4*. Group data analysis was performed with TEprepare as specified before, TEgroup_prepare and TEgroup_stats using the default parameters listed in table 3 and 4, and using the following custom parameters:

   for the function TEgroup_prepare we used:

cfg.shifttesttype = 'TEshift > TE';

   for the function TEgroup_stats we used:

% Design for statistical testing

% in cfg.design below

% first line (up to ;)

% - subjects for each dataset

% second line - corresponding

% condition for each dataset

%

% this is a dependend samples design

cfg.design =

[1,2,3,4,5,6,7,8,9,10,11,12,13,14,15,...

1,2,3,4,5,6,7,8,9,10,11,12,13,14,15;...

1,1,1,1,1,1,1,1,1,1,1,1,1,1,1,...

2,2,2,2,2,2,2,2,2,2,2,2,2,2,2];

% units of observation (subjects)

% are specified in line 1 of the design

cfg.uvar = 1;

% the value of the independent variable

% is specified in line 2

cfg.ivar = 2;

% Permutations within subjects,

% metric: t-value

cfg.permstatstype = 'depsamplesT';

% two-tailed testing

cfg.tail = 2;

##### Results

**Figure 8:**
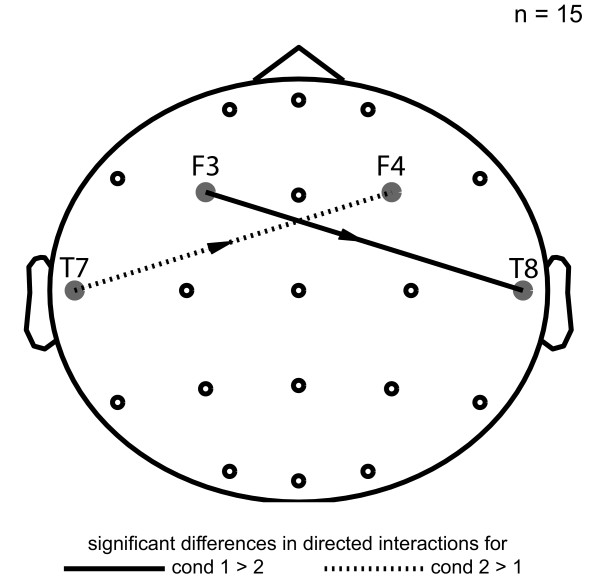
**Example of group data analysis**. Application example of group data analysis. In condition 1 a directed interaction from T3 to F4 and in condition 2 from F3 to T4 were simulated (for details see text). Solid arrow: condition 1 > condition 2; Dotted line: condition 2 > condition 1.

The simulated differences in directed interactions between both conditions were detected (FDR *q *< 0.05): *F3 *→ *T8 *for condition 1 (p-value << 0.001; t-value = 107.0160) and *T7 *→ *F4 *for condition 2 (p-value << 0.001; t-value = -114.9804) (Figure 8).

### Validation for neuronal data with known connectivity

When analyzing directed interactions in neural data, there is a plausible post-hoc explanation for any graph obtained because by far the largest part of neuronal connectivity is bi-directional in its anatomical structure. To circumvent this problem we chose a scenario where connectivity is known to be unidirectional. We recorded neuronal activity from the retina and the tectum of the turtle (*Pseudemys scripta elegans*), this way exploiting the fact the connectivity between the retina and the tectum is known to be unidirectional [41]. A second unidirectional connection in an information theoretic sense exists between the stimulating light source and the retina. A third, indirect one, exists between light source and tectum. All three of these connections are strictly unidirectional and together form an ideal test scenario for our purpose. For the sake of completeness we note that there are also backprojections from the brain to the retina in turtles. These *retinopetal *projections, however, are extremely sparse (some ten neurons) and do not originate in the tectum [42].

#### Preparation

Experiments were approved by the German local authorities (Regierungspraesidium, Hessen, Darmstadt). One turtle (Pseudemys scripta elegans) was anesthetized with 15 mg Ketamine, and 2 mg Medetomidinhydrochloride and decapitated. The entire brain with the eyes attached was removed as described in [43]. The brain was placed in a petri dish and superfused with oxygenated ringer. The ringer consisted of (in mM) 96.5 NaCl, 2.6 KCl, 2.0 MgCl2, 31.5 NaHCO3, 20 D-glucose, 4 CaCl2 at pH 7.4 and was administered at room temperature (22°C).

#### Electrophysiological recordings

The electroretinogram was recorded with a chlorided silver wire in a Vaseline well that was built around the right eye. The tectal signal was recorded in a superficial layer at the center of the left tectum with a quartz/platinum-tungsten electrode (Thomas Recordings, Giessen, Germany) with impedance 1 *M*Ω at 1 kHz. Data were amplified and filtered (1 Hz to 6 kHz) before being digitized at 32 kHz. For the analysis, data were low-pass filtered with 240 Hz, down sampled to 500 Hz and cut into 60 trials with 50 s each.

#### Visual stimulation

A sequence of red LED light pulses with random duration (uniform distribution between 1 ms and 2 s) and random inter pulse interval (uniform distribution between 1 ms and 5 s) was triggered via the parallel port using MATLAB and the Psychophysics Toolbox extension [44-46]. A light guide projected the full field flashes onto the retina.

#### Analysis settings

For the data preparation, we used TEprepare with its default values and the following specific parameters:

cfg.actthrvalue = 1000;

cfg.minnrtrials = 13;

cfg.maxlag = 15000;

cfg.predicttime_u = 16;

cfg.optimizemethod = 'cao';

and for the statistics we used TEsurrogatestats with its default values and the following specific parameters:

cfg.shifttesttype = 'TEshift > TE';

cfg.permstatstype = 'mean';

#### Results

**Figure 9:**
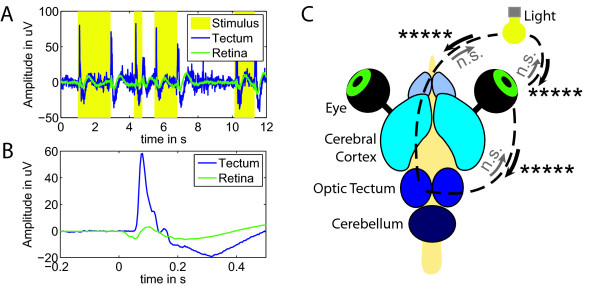
**Directed interactions in the turtle brain**. Directed interactions in the turtle brain. (A) Raw traces recorded in the tectum (blue) and from the retina (green) overlaid on the random light pulses (yellow). (B) The evoked responses for the tectum and the retina averaged to the onset of the light pulse (250 stimuli). (C) Turtle brain explant with eyes attached. Solid arrows indicate significant directed interactions (***** *p *< 10^-5^).

We found coupling from the ERG to the optic tectum (*p *< 10^-5^), but not for the opposite direction (Figure 9). We also found coupling from the light source time course to the ERG and to the optic tectum (*p *< 10^-5^), but not in the opposite directions (Figure 9) - in line with our expectations.

## Discussion

In this study we described the TE metric, its use in tests for the presence of directed interactions, and its implementation in TRENTOOL. Furthermore, we validated our implementation using simulated and real LFP data. From these simulations some important lessons can be learned that will be detailed in the following paragraphs.

The results of the first simulation (equations 14,15) demonstrate that the choice of the prediction time *u *^7 ^plays an important role for the detection of nonlinear interactions. In general, this necessitates a scan of potential values for the prediction time *u*, unless the interaction delay is known *a priori*. This scan is best performed on an independent set of pilot data. In this respect it is also important to note that both the false positive rate and the positive rate of the *shift test *were independent of our choice of *u *(see Figure 4A, *Y *→ *X*), such that the scanning procedure is not biased by false positives or false negatives due to shift-testing.

We also demonstrated the usefulness of the shift test for cases where instantaneous mixing is expected (equations 21-24, 25-28). These cases comprise, for example, EEG and MEG analyses at the sensor and the source level. In addition, scenarios where common noise potentially contaminates the measured signals also fall in this category because any kind of instantaneously shared signal or noise can increase false positive rates of measures based on Wiener's definition of causality [11,27,40]. Based on this, the shift test is recommended and performed by default in TRENTOOL.

### Comparison to other methods and toolboxes

A researcher interested in the estimation of directed interactions in neural data is faced with a decision to use either model based tools or a model free analysis based on the TE metric proposed here. Model-based tools comprise Dynamic Causal Modeling (DCM) [47-49], based on neural mass models, or Granger causality tools [50,51] based on linear signal and interaction models ^8^. TE analysis and DCM complement each other as the first serves an exploratory purpose while the latter is used to validate a model by comparing it to alternatives [52]. In contrast, the relationship between TE and linear Granger causality sometimes caused confusion. Indeed, both approaches are equivalent for Gaussian data [16]. However, neural data are often non-Gaussian as demonstrated by the validity of independent components extracted from neural data based on their non-Gaussianity (see for example [53]). Furthermore, non-Gaussian independent components from EEG data correlate well with those extracted independently with fMRI-constrained source modeling [54]. Thus, a restriction to Gaussian data models alone is suboptimal if the exploration of a model space as large as possible is the goal of the analysis.

To our knowledge there exist three other publicly available toolboxes or libraries for computing TE: NTE (http://chelly.us/lab/transfer_entropy), the MATLAB TE toolbox (current version 0.2; http://code.google.com/p/transfer-entropy-toolbox/), and TIM (http://www.cs.tut.fi/~timhome/tim/tim.htm). In contrast to TRENTOOL, the former two toolboxes target the computation of TE from sparse binary time-series, instead of analog signals. TIM and TRENTOOL indeed share the goal of estimating TE from analog time series, TIM however, does not provide a complete statistical framework for significance testing in neural data, and to our knowledge no equivalent of a shift test. Another important benefit of TRENTOOL compared to other TE estimation tools is the inclusion of various optimization routines for the choice of the embedding parameters, i.e. the embedding dimension d and the embedding delay *τ *[32,34]. The choice of the correct or best values for these parameters is not obvious and trivial, but has far reaching consequences - as detailed in the implementation section. Here, we demonstrated these consequences by a dimension scan, where the best results were found with the optimal embedding parameters estimated by the parameter optimization algorithms (Figure 5). With these optimal embedding parameters, TRENTOOL was able to find the information flow between two signals even in the presence of a high level of noise (white noise of up to 200% of the original signals' variance).

Although TE is a powerful tool for exploratory data analysis it has some practical limitations, most of which are generic to connectivity analysis (see [11,27] for a detailed discussion). Perhaps the most important limitation of TRENTOOL - but not of TE in general - is its current limitation to bivariate analyses. This fact must be taken into consideration when interpreting results, and can be mitigated by subsequent confirmatory, model based analyses that allow for nonlinearities - such as DCM. As described below, several approximative techniques to provide a multivariate estimation from limited data are investigated at the moment to overcome these problems.

### User-friendliness and open source concept

Although TRENTOOL does not provide a graphical user interface, TRENTOOL aims to be user-friendly and make the computational methods available for experimental studies. Since TRENTOOL analyses are based on MATLAB scripts, documentation of all relevant analysis parameters is straightforward and the interaction between students and supervisors can be based on this documentation. TRENTOOL analysis scripts typically comprise just two or three high level functions and the specification of a handful of analysis parameters. Therefore the required programming skills of a potential TRENTOOL user are not much different from the basic building blocks needed for one of the established EEG and MEG (or other brain imaging techniques) analysis toolkits (e.g., shell scripting for AFNI and FSL command line tools, or MATLAB-scripting of FieldTrip or SPM functions). Despite the simple usage, the open source nature of the toolbox allows the researcher interested in understanding and extending the method to examine the implementation in detail.

From a programmers point of view TRENTOOL is closely related to FieldTrip [28]. For the use with neural data like MEG, EEG and LFP data, TRENTOOL seamlessly integrates with this popular toolbox by sharing a common data format.

### Application scenarios

Despite its integration with FieldTrip, TRENTOOL is not limited to neural data. Anywhere where two interacting time series can be measured, it is possible to use TRENTOOL to analyze them (e.g. dynamics of the stock market, wave motion in oceanography and audiography). We designed TRENTOOL as an open source toolbox, because this gives maximum control to users and developers. Everyone can see the code, learn from it or change it to accommodate their individual needs, spawning new applications.

### Future perspective

As the limitation to bivariate analysis is the most important limitation of TRENTOOL we are working on multivariate extensions, using either multivariate TE formulations (see e.g. [29]) or techniques based on the identification of interaction delays. Further releases of TRENTOOL will also extent the currently available functionality. Some features that will be included in future releases are the application to fMRI data, and an extended range of accepted input formats. Another goal of development is the inclusion of TRENTOOL in the FieldTrip distribution in the near future

## Conclusion

Transfer entropy is an information theoretic implementation of Wiener's principle of causality. It offers an approach to the detection of neuronal interactions that is free of an explicit model of the interactions. Hence, it offers the power to analyze linear and nonlinear interactions alike. This allows the comprehensive analysis of directed interactions in neural networks at various levels of description. Here we present the open-source MATLAB toolbox TRENTOOL that allows the user to handle the considerable complexity of this measure and to validate the obtained results using non-parametrical statistical testing.

## Notes

^1 ^Notable exceptions within Wiener's framework are the work of Freiwald and colleagues who used a nonlinear approach [55] and of Leistritz and colleagues who further relaxed modeling assumptions by using self-exciting autoregressive threshold models and allowing state dependence of the modeling parameters [56]. Model free nonlinear prediction schemes were also used by Terry and Breakspear in their analysis of EEG data [57], based on earlier work by Pecora [58].

^2 ^See discussion section for toolboxes or libraries that provide general TE estimation.

^3 ^Numerical TE values in the output may be negative, due to bias - see [38] for more details.

^4 ^There are two ways in which a suboptimal choice for *τ *may compromise predictions: If *τ *is too large, the embedding vector might include successive independent elements and therefore create a too homogeneous distribution in the reconstructed state space. If *τ *is too small the embedding vectors will include highly correlated elements and produce clusters around the diagonal in the state space. In either case a meaningful neighborhood cannot be found.

^5 ^An even more conservative test would be to demand positive evidence against volume conduction, i.e. values for *TE*_*i*_(*X *→ *Y*) that are significantly larger than for *TE*_*i*_(*X' *→ *Y*). This behavior is also implemented in TRENTOOL and can be switched via input configurations.

^6 ^Users can find tools for their own simulation on the TRENTOOL Homepage at http://www.trentool.de/ARSimTool.zip

^7 ^The prediction time *u *is not an embedding parameter but a parameter of our specific estimator, see equation 5.

^8 ^Wiener's formalism of increased predictability is not limited to linear implementations - see for example [6].

## Availability and requirements

• Project name: TRENTOOL (TRansfer ENtropy TOOLbox)

• Project home page: http://www.trentool.de

• Operating system: Platform independent

• Programming language: MATLAB (toolbox tested on R2008b and successive releases) and C

• Other requirements: The following software is necessary to run TRENTOOL: MATLAB 7.4 or higher with statistic toolbox (http://www.mathworks.com/), TSTOOL (http://www.dpi.physik.uni-goettingen.de/tstool/), FieldTrip ([28], http://www.ru.nl/neuroimaging/fieldtrip)

• License: GNU GPL v3

• Restrictions: There are no restrictions on academic or commercial use of GPL v3 software as long as the restrictions of the GPL v3 license are respected. For academic use we would appreciate a citation of the current publication.

## Abbreviations

ACT: autocorrelation decay time; AR(10): stable autoregressive processes with order 10; *d*: embedding dimension; EEG: electroencephalography; ERG: electroretinogram; ε: mixing coefficient; *η*: Gaussian white noise process; FDR: False Discovery Rate; fMRI: functional magnetic resonance imaging; LFP: local field potential; MEG: magnetoencephalography; MI: mutual information; *τ*: embedding delay; TE: Transfer entropy; TRENTOOL: TRansfer ENtropy TOOLbox; *u*: prediction time

## Authors'contributions

ML programmed and co-designed the toolbox, generated and analyzed the simulated data and wrote the paper. RV conceived and co-implemented the transfer entropy algorithm presented here and wrote the sections on the definition and computation of transfer entropy. VP recorded and analyzed the Turtle ERG and LFP data and co-wrote the manuscript. MW conceived of the project, invented the shift-test, designed the toolbox and statistics routines, co-implemented the TE algorithm and co-wrote the paper. All authors read and approved the final manuscript.

## Supplementary Material

Additional file 1 **This article comes with a zip archive of the most recent version of TRENTOOL at publishing of this article**. It is strongly recommended to check http://www.trentool.de for newer releases and updated documentation. Click here for file
